# How and why should we implement genomics into conservation?

**DOI:** 10.1111/eva.12193

**Published:** 2014-08-19

**Authors:** Barry J McMahon, Emma C Teeling, Jacob Höglund

**Affiliations:** 1 UCD School of Agriculture & Food Science, University College DublinBelfield, Dublin 4, Ireland; 2 UCD School of Biology & Environmental Science, University College DublinBelfield, Dublin 4, Ireland; 3 Department of Ecology and Genetics, Evolutionary Biology Centre, Uppsala UniversityUppsala, Sweden

**Keywords:** adaptation, conservation, conservation prior, genomics, next generation sequencing

## Abstract

Conservation genetics has provided important information into the dynamics of endangered populations. The rapid development of genomic methods has posed an important question, namely where do genetics and genomics sit in relation to their application in the conservation of species? Although genetics can answer a number of relevant questions related to conservation, the argument for the application of genomics is not yet fully exploited. Here, we explore the transition and rationale for the move from genetic to genomic research in conservation biology and the utility of such research. We explore the idea of a ‘conservation prior’ and how this can be determined by genomic data and used in the management of populations. We depict three different conservation scenarios and describe how genomic data can drive management action in each situation. We conclude that the most effective applications of genomics will be to inform stakeholders with the aim of avoiding ‘emergency room conservation’.

## Introduction

The last few decades have seen the emergence of genetics as a tool in conservation research and conservation management, and a new field of science and conservation genetics has arisen (Allendorf and Luikart 2007). Conservation genetics has provided important insights into the dynamics of endangered populations. In particular, it has facilitated empirical insights into how the process of inbreeding and increased genetic drift leads to loss of genetic variability in small, isolated populations (Höglund 2009). This has been facilitated through the development of molecular tools such as analyses of genetic variation at allozyme loci and at the level of DNA: loci coding for organelle and nuclear DNA, microsatellites and amplified fragment length polymorphisms (AFLPs). Additionally, conservation genetics has made important contributions by translating data on neutral makers into interpretations on metapopulation structure, gene flow, demography, effective population size and evolutionary history (see below, Höglund 2009; Puechmaille et al. 2011).

With the recent advancements in genetic tools and technologies, another new research field has emerged: genomics. In this paper, we use the term ‘genomics’ broadly to refer to data and downstream analyses generated by next generation sequencing (NGS) techniques. Thus, we include techniques such as transcriptome sequencing, data from reduced sequencing libraries, and typing of SNPs called by NGS techniques as well as strict whole-genome approaches under this term. Today, it is possible to gather massive amounts of genetic data not only from the so-called genetic model species, for example the mouse, *Mus musculus*, the weed *Arabidopsis thaliana*, the fruit fly *Drosophila melonogaster*, the nematode *Caenorhabditis elegans* and man which have been subject of intensive genetic research during the last half century (Genome 10K 2009; Perry et al. 2012a). Today, any species can be sequenced at moderate cost and effort, and orders of magnitude more genetic markers compared with previously can be studied and used in applied research like conservation (Ekblom and Galindo 2011; Gayral et al. 2013).

With the advent of genomics, an important question emerges: should genomic tools and whole genomes be used in conservation studies? Genomics offer lots of promises, and the conservation genetics community have been enthusiastic about its prospects for some time (Allendorf et al. 2010; Avise 2010; Ouburg et al. 2010; Funk et al. 2012). Yet, there are very few concrete examples of where genomics have made a major impact. Thus, why is the change from genetics to genomics not happening faster? In this paper, we argue that this is partly due to the fact that the older genetic methods may still provide satisfactory answers to many conservation questions. The estimates of, for example, parameters such as population structure, migration rates and effective size can be quite accurately estimated with the use of the old tools (e.g. a multilocus microsatellite study). With more data, parameter estimates will, of course, be more accurate, but the issue is whether this marginal improvement is worth the increasing costs. So, why bother with genomics? Is the deluge of data worth the effort? Why should we generate so much data when simple Sanger sequencing methods and downstream analyses may provide the information we need for conservation?

There are two unsatisfactory answers to the above questions and one potentially acceptable answer. The first unsatisfactory answer to the question posed above may be: because we can. Genomic techniques become cheaper and more accessible by the day and can in principle be applied to any species (McCormack et al. 2013). We argue that applying the new and still more costly techniques are likely to only marginally improve the above mentioned parameter estimates that can be obtained with the old tools. Because we can is thus not a good answer, and genomics should not be used unless a specific argument for its use can be put forward.

Another unsatisfactory answer may be: because we are forced to. Conservation is not and will not be driving the methods development in the field of genetics. The development of new methods and equipment will instead be driven by the needs and possibilities in fields like human genetics, biomedical research and plant and animal breeding. These fields are where the big markets for biotechnology lie, and this will be so also in the future. Conservationists inclined to use genetic and genomic tools can take advantage of the advances but are unlikely to contribute substantially to new sequencing techniques and methods (McCormack et al. 2013). Here, conservation geneticists are in a similar situation to those who work on ancient DNA. We can endorse the technical advances and take advantage of them when applicable, but we are faced with the problem that the new methods often are not specifically designed to our needs (McCormack et al. 2013). Another related issue is that as general genetics make advances, the biotech companies that we now rely on to provide us with equipment are unlikely to develop and maintain the old machines and chemistries we have been using. Anyone who has been in a genetics lab for some time can just think back to the techniques and equipment we were using 5, 10 and 15 years ago. Some of the studies that were performed then are simply not possible to do today unless the old equipment is still in shape and running, and typically not feasible without expensive replacement of discontinued parts. Another difficulty with conservation genetic research using traditional markers is that now more and more journals require large amounts of sequence/SNP data from whole genomes for publication, even if the question can be adequately addressed with data from, say 12 loci microsatellite analyses, thus forcing all labs to embrace the new genomic technologies. Therefore, because we are forced to is thus a relevant but rather unsatisfactory answer.

Lastly, another and perhaps better answer to why bother with genomics is that geneticists are still debating what parts of the genome are important for principal evolutionary processes like speciation, adaptation to local conditions and hence species ability to survive in a changing world (Puechmaille et al. 2011; Perry et al. 2012a,b; Orlando et al. 2013). Genetic, and hence genomic, diversity is recognized as one of the most fundamental levels of biodiversity [along with species diversity, community diversity and ecosystem diversity (Genome 10K 2009)]. One lesson from the genomic studies so far is that genomes vary enormously in diversity within species (1000 Genomes Project Consortium 2010). Large parts are invariant (even among species), while other regions are hypervariable, like the genes in the major histocompatibility complex (MHC), a group of genes coding for genes involved in the immune defence of vertebrates (Acevedo-Whitehouse and Cunningham 2006). This fundamental issue, in which parts of the genomes are important for species survival, still begs an answer and still remains a grand challenge in Biology.

Applying genomics to conservation studies is the only way forward here. What should be conserved and what matters most in order for species survival: neutral or adaptive variation? This can only be solved with more genetic data and genomic techniques (Allendorf et al. 2010). In order to study this issue, the genomes of endangered species need to be sampled at a much higher density and with more markers than what has typically been the case in traditional conservation genetic studies where typically 10–20 loci of microsatellite markers have been considered to a good number of sampled loci (McCormack et al. 2013). Therefore, despite the difficulties in generating, analysing and managing genomic data, it is imperative for the future of conservation science that researchers embrace these new technologies and modify to purpose.

There are arguably other applications where the use of genomic tools may become relevant such as estimating past and present demographic parameters, understanding genetic diseases, the molecular basis for inbreeding, phylogenetic issues and detecting hybridization/introgression (Fitzpatrick et al. 2012; Miller et al. 2012). Genomics will certainly contribute to these areas as well, but these issues are not confined to case studies of endangered species and populations, and hence, the applicability of genomics in these respects will probably best be addressed by general ecological genomic studies outside the restrictions imposed by a given conservation context, although the outcome is of course still relevant for conservation.

Although there are number of published reviews on the pros and cons of genomics in conservation (e.g. Luikart et al. 2003; Allendorf et al. 2010; Avise 2010; Ouburg et al. 2010; Funk et al. 2012; Hoban et al. 2013), genomic studies with a clear conservation aim are still relatively rare. The genome of the giant panda was published in 2010 (Li et al. 2010), and the bonobo and chimpanzee genomes have been compared with the human genome (Prufer et al. 2012). Likewise, the whole genome of the Madagascar lemur aye-aye was published in 2012 (Perry et al. 2012b). These three studies were descriptive papers which provided valuable genomic resources useful for conservation, although neither paper addressed a specific conservation issue. Recently, whole-genome SNP data were used to infer demographic history and conservation units in the great apes (Prado-Martinez et al. 2013), and NGS-generated SNPs helped to uncover the subspecies status of *Pan troglodytes ellioti* when population structure was conflicting when using microsatellites (Bowden et al. 2012). A simliar case can be made for forest and Savannah elephants where NGS data have helped to reveal a deep speciation event in African elephants (Rolandh et al. 2010). Genetic clustering analyses using 44.000 SNPs revealed population structure and inbreeding structure among North American wolf populations of conservation concern (Von Holdt et al. 2011). Whole-genome sequencing of giant pandas have provided valuable insights into demographic history and local adaptations in giant pandas (Zhao et al. 2013). Similarly, whole-genome data on two endangered falcon species have shed light on the divergent demographic histories and the predatory life style of two species of falcon (Zhan et al. 2013), and the whole genome of the black grouse has been compared with domesticated chicken and turkey providing evidence for faster sex chromosome and MHC evolution (Wang et al. 2014).

## Identifying conservation priors

At present, the world is losing species at a rate comparable to the mass extinctions signifying the major transitions of geological time periods (Butchart et al. 2010). Previous mass extinctions can be attributed to geological and extra-terrestrial impact, while the present mass extinction is caused by human impact (Wilson 2001). Society has to find means to counteract this loss of biodiversity and save habitat and areas where threatened species reside. This will be economically costly. To effectively conserve, such areas and habitats could cost US$ 76 billion a year, while the cost of protecting all the world's threatened bird species is estimated at US 0.875 to US$ 1.23 billion a year (McCarthy et al. 2012). It is important to preserve species and endangered populations despite costs, but pragmatism in conservation is vital. In addition, conservation must look beyond and aspire to more than simply ‘conservation life support’ or conservation at the ‘emergency room door’ (Redford et al. 2011). In relation to policy decisions regarding wildlife conservation and management, a simple set of questions that relate to the issue should be initially addressed to focus the decision-making process, particularly in relation to feasibility. These questions can be used to formulate the conservation prior related to the issue. A composite set of such questions adopted from Sinclair et al. (2006) is outlined below:

1. Where are we now?
2. Where do we want to go?
3. Can we get there?
4. Will we know when we arrive?
5. How can we get there?
6. What are the disadvantages?
7. What benefits are gained?
8. Are benefits greater than the disadvantages?

For the purposes of this paper, a conservation prior is defined as a specific predetermined objective that aims to enhance and improve the viability of a population or species. This includes populations or species that face imminent extinction but should also aim for prophylaxis in this regard. In relation to devising the conservation prior, firstly, one should question what the goal of the conservation initiative is and ultimately determine what specific end point should be achieved. Next, the conservation methods to be employed should be determined, eventually choosing the methodologies best suited to the budget and ultimate conservation goal. Finally, the probability that the proposed specific conservation objective will be successful should be elucidated taking past endeavours into account (Sinclair et al. 2006). We argue there is a role and need for conservation genomics to identify the conservation prior.

Addressing the above three steps will enable the ‘conservation prior’ to be determined and will establish which actions are most likely to lead to a successful outcome. This should routinely be undertaken by the conservation managers and those directly involved in implementing the management decision. Conservation activities that have increased probability of success should be prioritized. The identification of conservation priors is fundamental as there are limited resources and numerous species regionally, nationally and globally that require conservation and management intervention. How can such conservation priors be decided upon, what are the data needed and what are the tools at hand to guide conservationists to optimal resource allocations? Below, we describe scenarios where genomics may become useful in identifying the conservation prior.

## Scenarios where genetic data can be informative

Both genetic and genomic techniques can inform managers of the presence of inbreeding depression, population structure, effective population size and whether or not populations are isolated and fragmented (Höglund 2009). While some of these applications may be improved by the shift from genetic to genomic data, this is not likely to change interpretations and conclusions more than marginally. It is therefore important that the scientific community recognizes where genomics may play a different role than what has been possible with the traditional conservation genetic methods. We argue below that genomics may provide a cost-effective means of assessing relevant genetic diversity and thus the conservation status for threatened species.

If the conservation prior is to prevent global or local extinction, conservation efforts are required at the population level (e.g. black grouse *Tetrao tetrix*, Ethiopian wolf *Canis simensis*, Californian condor *Gymnogyps californianus*), and in such cases, the conservation efforts could be guided or at least informed by genetic data. The applications of the findings can be used to identify viable/nonviable populations. These data can provide critical information that population census data may mask, for example when comparing two sets of population census data, a population which is numerically greater may have relatively less heterozygosity than smaller population which has greater heterozygosity and therefore could have a great chance of survival in the medium to long-term, thus allowing wildlife managers implement a more informed set of actions. However, it is imperative that scientists make it more understandable, and wildlife managers need to respect the potential the field may have in regard to the conservation of populations.

Outlined below are a set of scenarios with species specific examples of the application of genetics for assessing the conservation prior which could potentially be improved by the acquisition of appropriate genomic data. These scenarios range from critically endangered populations to monitoring gene flow and genetic drift in large contiguous vibrant populations (Table1).

**Table 1:** Outlines the framework for conservation action given information regarding the genetic integrity of populations.

A framework for conservation genomic actions			
Demographic situation	Approximate effective size, *N*_e_	Genetic problem	Conservation Action
1. Isolated populations with <30 individuals, for example Black grouse in the Netherlands, Florida panther	<10	Low variation	Increase variation by artificial augmentation or translocations
2. Fragmented meta-populations with up to one hundred individuals, for example Irish red grouse	<100	Genetic divergence, increased drift or possible local adaption	Increase gene flow among local units if drift, preserve local ecotypes if local adaptations
3. Populations with large contiguous populations, for example Scandinavian willow grouse	>100	None	Observe and monitor

### Scenario 1

In a situation where the effective population size is very low (<10), for example Dutch black grouse (Larsson et al. 2008), this is invariably associated with isolated, fragmented populations signified by low genetic variation. The most important evolutionary forces in this situation are genetic drift and inbreeding, both leading to rapid loss of genetic diversity. Translocations or population augmentations, if advisable and desirable, may be the only way to save such populations from extinction, and issues about local adaptation are thus of minor importance, simply because stochastic processes are much more important than selection in very small populations. This situation may be referred to as ‘conservation life support’ as removal of conservation efforts will plausibly result in extinction of the population.

### Scenario 2

Where the approximate effective population size is slightly larger but still small (<100), for example Irish red grouse (McMahon et al. 2012), genetic divergence among discrete populations may exist, but a process of increased drift has occurred interfering with possible local adaption. In many respects, this may be the situation where conservation genetics or in the future genomics is most informative as concealed information can be obtained regarding the viability of existing fragmented populations and the presence of locally adaptive single nucleotide variations correlating with higher survival rates; for example, levels of heterozygosity may not be directly related to present census population size. In this situation, it may be most cost-effective from an economic perspective to identify the most viable populations and identify and remove possible barriers to gene flow as slipping into ‘conservation life support’ (scenario 1) is usually very expensive. Depending on the result of the population studies as to whether there is local adaptation or not, this will influence the conservation action to increase gene flow or to preserve local ecotypes.

### Scenario 3

The situation where the effective population size is large (>100), for example Scandinavian willow grouse (Berlin et al. 2008), is a good position for the conservationists as this situation is there is likely to be associated with a large contiguous viable population. The required action is to observe and monitor the population, as there is no need to currently intervene. Such populations may also be allowed to be managed a culled in a sustainable manner.

A specific conservation prior should be used to avoid a population slipping from scenario 2 to scenario 1 as this will result in ‘conservation fire fighting and life support’ which, by its very nature, is economically costly and ecologically difficult. Conservation genetics and, in the future, conservation genomics may be utilized in this particular situation to inform managers of the viability of a population prior to adverse symptoms manifesting themselves in population declines. Conservation genomics will be useful in identifying the loci and genomic regions responsible for adaption to local conditions and will help identifying ecotypes of conservation value. This can inform managers whether translocation to relatively threatened populations from elsewhere is a viable conservation strategy. In addition, these methods could be utilized as monitoring tools under scenario 3 providing information on the population and further enhancing knowledge regarding the molecular contribution to population and dynamics regulation.

## From genetics to genomics

According to a recent meta-analysis, the ecological inference from molecular ecology studies has been limited (Johnson et al. 2009), and there is a risk that genomic studies would be even further away from ‘real world conservation’. In the scenarios outlined above, many of the issues can be addressed with traditional techniques. So, how can genomic data inform the conservation prior better? How do researchers and managers working on a daily basis in conservation and management of threatened species harness genomic techniques to clarify problems and facilitate the effective management of species and populations?

As argued above, we think that ‘local adaptation’ is the most important issue where genomics can contribute to conservation science. We want to stress that we do not see ‘local adaptation’ as different from the issue of ‘preserving genetic variation’ or ‘identifying ecotypes’. These aspects are instead tightly linked. Without genetic variation, there can be no local adaptation, and without local adaptation, no ecotypes. Further, simply because local adaptation is the most important aspect, this does not exclude, for example ‘estimation of demographic parameters’. Our argument is simply that the first is more important, not that the second is unimportant.

Theoretical population genetics have in the recent decades seen a shift in how population genetics is modelled and analysed. In the classical Fisher–Wright models of population genetics, allele frequency change was modelled as a forward in time process. With the advent of more and more sequence data, theoretical population geneticists have started to think about population processes in reverse time, and empiricists are now more and more analysing data using coalescent approaches (Hein et al. 2005; Wakely 2008). Because of recombination, a diploid genome will have parts that coalesce at different times, and moreover, these independently segregating parts of the genome are independent samples reflecting the demographic history of the species (Li and Durbin 2011). Coalescent-based analyses have therefore called for a shift in how genetic samples are obtained. In the past, there was a focus on one or a few genes which required sampling of many individuals. With modern coalescent approaches, it is not the number of individuals sampled that matters but the number of genes. At one extreme, the entire genome from a single individual will provide more than enough data for many kinds of analyses. Recent studies of prehistoric human populations have shown that large-scale genome analyses of a few individuals may provide data for inference of past demographic history and provide good estimates of genetic variation (e.g. Schlebusch et al. 2012). Applying genomics to conservation will thus mean going from ‘one gene, many individuals’ to a ‘few individuals, several genes’ approach. When conservation biologists and stakeholders realize this, sampling and monitoring programs need to be changed accordingly. While the lab costs for a genomic may study still supersede the lab costs for traditional conservation genetics, a less labour intensive sampling may cut the costs of the conservation project on the nonlab part of the project. The ‘many genes one individual’ approach is of course an extreme, and in reality, pooled population resequencing with shallow coverage may become the most widely adopted approach (see e.g. Jones et al. 2012; Lamichhaney et al. 2012).

With the appropriate facilities and collaborations, genomic information can be collated on a species or population that will inform a conservation strategy for the population. In addition, a key component to the application of conservation genomics data is full comprehension of what the data represents and that this data will be more difficult to understand than genetic data principally because it will be gathered in enormous quantities. There will thus be a need for handling genomic data and to effectively use genomics with a specific aim to inform conservation action. Perhaps genomics has not yet exploited the field of conservation (Segelbacher and Höglund 2009), but more importantly, conservation may not have explored the full potential of genomics and the questions that these techniques can answer. Conservationists need to at least understand the basics of what the genomic techniques can tell us, and results of analyses need to be examined in conjunction with other ecological factors in order for efficient conservation. Importantly, categorization of conservation status must continue to include other factors, as is the case with species classification, for example land use changes and projected future declines. Extinction of a population is usually caused by a range of activities that can cause the population to cascade into an extinction vortex, but information on the genomics can be a critical in order to assess the viability of a population.

The most effective applications of genomics are to help managers and ‘stakeholders’ to as much as possible avoid ‘emergency room conservation’. Such a scenario could be where there are species and populations in scenario 2 but drifting towards scenario 1 (Table1). If there is intervention, an acquisition of population data on the genomic level in conjunction with other relevant ecological information an action plan could be operationalized to halt the declines of the population (Ryder 2005; Romanov et al. 2009). This could prevent scenario 1 where there is large-scale conservation intervention that is costly and running a large risk of failure (e.g. Californian condor and Dutch Black Grouse). The overall philosophy of the conservationists and managers should be to observe and carry out noninvasive or nonintrusive actions. Conservation policy should aim to avoid a situation where translocations are required; rather, focus should be placed on the implementation of appropriate conventional methods that will prevent conservation life support. The overall goal of any long-term sustainable conservation policy is to allow populations that are large enough to harbour enough genetic variation so that they can respond and evolve in response to ever-changing selective pressures. Wildlife conservation and management personnel should be aware of the capacity of genomics techniques available to them and harness all information that will enable them to carry out more effective conservation.

## How to implement genomics into conservation?

In studies of many threatened and endangered wild populations, a restriction is put on the interference allowed with such populations. It is often not advisable to subject individuals in such populations to the stress and risk of harm induced by catching and handling them, and thus, conservation genomic studies may often have to rely on low-quality noninvasively sampled DNA. A genomic study would require at least, at some stage, access to rather large quantities of high-quality DNA. How should a feasible conservation genomic study, given the potential limits of DNA collection in endangered populations, be designed? We have argued that an important first step is to identify the conservation prior. What is the goal of the study and what issues do we need to solve in order to devise a successful conservation programme? Genomics should be employed but must be as cost-effective and designed in the best way possible to address a specific set of scientific questions. We have argued that when it comes to identifying the genetic background and loci involved on the formation of ecotypes and in studies of local adaptation, genomics is an unprecedented approach and offers possibilities that the older techniques cannot.

If it is decided that a genomic study is of value, there are a number of ways and competing techniques on how to approach the goal. It is beyond the scope of the present paper to review these techniques and analytical tools, such as whole-genome sequencing, RNA-seq, restricted-site associated DNA-sequencing (RAD), SNP typing and genome-wide association studies, and details on how to go about will be covered by relevant papers in this issue. Below, we instead provide some general thoughts and guidelines.

Past studies of the black grouse may serve as a useful example. This species is numerous and not threatened in the north and east of its distribution, while populations in western Europe are of considerable conservation concern and can be found in a range of situations: from very small and completely isolated to isolated but rather large (Höglund 2009). In this species, it is thus possible to compare genetic variation in populations under a range of demographic scenarios and threat status (Höglund 2009). It has also been possible to reconstruct genetic variation in the recent past using genetic profiles from museum specimen (Larsson et al. 2008; Strand et al. 2012).

A range of molecular markers have been applied to address issues of population genetic diversity (heterozygosity) (Höglund et al. 2007; Strand et al. 2012), inbreeding (Höglund et al. 2002; Larsson et al. 2008), gene flow/isolation (Höglund et al. 2011; Svobodova et al. 2011; Corrales and Höglund 2012) and postglacial expansions (Corrales et al. 2014). An important lesson concerning genetic diversity estimates is that while the prediction of lower genetic diversity in smaller and fragmented populations as compared to large outbred ones is met in studies of microsatellite loci and SNPs, the pattern is less obvious when it comes to studies of hypervariable MHC loci (Strand et al. 2012). In the latter case, genetic diversity may simply remain because drift has not yet had time to erode the previously unusually high levels of allelic diversity found at MHC loci. This illustrates the importance of knowing the genomic position and function of the loci studied (Wang et al. 2012a; Strand et al. 2013). Although neutrality tests often fail to reject the neutrality of microsatellite loci, it would be most useful to go from anonymous markers, such as those developed by Piertney and Höglund (2001), to markers with known chromosomal position and gene association.

One way to achieve this is to sequence a transcriptome (Wang et al. 2012b) or a draft genome (Wang et al. 2014) from a few individuals of the target species. For such analyses, one would need large quantities of high-quality RNA/DNA, and if such cannot be sampled in the target population, perhaps there alternative sources such as individuals in a more viable population of the same species or a zoo or a botanical garden. With the aid of bioinformatic techniques, such as a reference guided assembly, it is possible to assemble a useful draft genome where microsatellite loci within UTR's of known genes and SNPs with known chromosomal position can be identified. These ‘un-anonymous’ markers can later be applied for typing a larger number of individuals using noninvasively sampled DNA. Here, SNP-typing methods will be very useful because many of the typing techniques can handle fragmented DNA in low quantities. Even without a draft genome, it is possible to design studies using genomic techniques at moderate costs such as RAD (McCormack et al. 2013), but this requires better quality DNA and genotyping by sequencing randomly amplified regions using protocols akin to AFLP (Parchman et al. 2012). These latter methods would increase the number of markers, but without a reference genome, the markers would still be anonymous.

What is conservation genetics and what is conservation genomics? It should be clear from the above that the two fields are not separate entities but are highly integrated and interdependent. As an example, consider SNP mining and population structure in grouse (Höglund et al. 2013) and hake (Milano et al. 2014). Both studies started by sequencing genes and detecting SNPs to elucidate population structure. The grouse study used genomic information from chicken and sequenced a number of candidate genes with Sanger sequencing. The hake study used RNA-seq to detect SNPs with more modern NGS techniques. The grouse study found 127 SNPs, and the hake study 381. The latter is told as a ‘genomic study’, while the first one is not, but in essence, the two studies are very similar as both address important conservation questions, demonstrating that conservation genetics and conservation genomics techniques are not mutually exclusive.

## Conclusions and perspectives

We predict that genomics will make a difference primarily in determining which parts of the genomes are responsible for local adaptation and therefore important to preserve. These are the genetic aspects of a conservation programme, but genomics may also aid in more ecological investigations such as estimating population size. Here, genetic techniques have not only been used to estimate the effective size of populations (i.e. genetic variability) but also ecological population size using genetic profiles of remains like scats and feathers with mark-recapture calculations without actually catching the individuals (Puechmaille and Petit 2007). Genetics have also provided cost-effective alternatives to costly and labour intensive radio and satellite tracking in estimating movements and dispersal (e.g. Fabbri et al. 2007; Sahlsten et al. 2008). Studies such as these will be, as argued above, improved by genomic data but not dramatically changed. However, an area in which genomic data will be particularly helpful is in attempts to estimate past demographics such as population size fluctuations. Here, the old genetic techniques fall short because with few markers, it is difficult to disentangle demographic events such population size bottlenecks from selection. However, genomic data, even from a single individual, can provide useful insights in an endangered species past population dynamics (Zhan et al. 2013). This would allow us to elucidate whether endangered species have been endangered and bottlenecked also during their past evolutionary history or whether their present threat status is a consequence of what is currently happening therefore providing information as to how the situation can be rectified. Conservation biology should now embrace the new field of genomics and engender meaningful discussions between managers and scientist to ultimately avoid the deleterious and costly effect of ‘emergency room conservation’.
